# Effects of GLP1RAs on pregnancy rate and menstrual cyclicity in women with polycystic ovary syndrome: a meta-analysis and systematic review

**DOI:** 10.1186/s12902-023-01500-5

**Published:** 2023-11-08

**Authors:** Lingling Zhou, Huanjia Qu, Lu Yang, Lan Shou

**Affiliations:** Metabolic Disease Center, Department of Endocrinology and Metabolic Disease, Affiliated Hospital of Hangzhou Normal University, Hangzhou Normal University, Hangzhou, China

**Keywords:** Polycystic ovary syndrome, Glucagon-like peptide 1, Pregnancy rate, Menstrual patterns, Meta-analysis

## Abstract

**Purpose:**

This study was aimed to assess the effectiveness of Glucagon-like peptide 1 receptor agonists on pregnancy rate, menses, anthropometric and hormonal parameters in PCOS patients.

**Methods:**

We conducted searches of the published literature in PubMed, EMBASE, Cochrane Library, Web of Science up to September 2022. Data from randomized controlled trials were obtained to assess the effects of GLP1RAs in PCOS women. Weighted mean difference, standardized mean difference, and risks ratio were employed for effect size estimation using a random-effects model.

**Results:**

A total of 840 patients with 469 individuals in GLP1RAs group and 371 individuals in control group from 11 RCTs were included. GLP1RAs usage was associated with an improvement in natural pregnancy rate (RR: 1.72, 95% CI 1.22 to 2.43, *P* = 0.002, *I*^2^ = 0%) and menstrual regularity (SMD: 1.72, 95% CI 0.60 to 2.85, *P* < 0.001, *I*^2^ = 95.6%). There were no statistically significant differences in total pregnancy rate, IVF pregnancy rate between two groups, but total PR elevated in a short time after GLP1RAs as shown in subgroup analysis. Randomization to GLP1RAs treatment was associated with great improvement in HOMA-IR, BMI, WC, SHBG and a slight reduction in TT compared to control group. A decrease in TBF was seen in European population. GLP1RAs monotherapy was not superior to metformin when it came to fT, DHEAS, FAI.

**Conclusions:**

Prescription of GLP1RAs improves natural pregnancy rate, menstrual cyclicity and insulin sensitivity, anthropometrics, hormonal indexes in PCOS women.

**Supplementary Information:**

The online version contains supplementary material available at 10.1186/s12902-023-01500-5.

## Introduction

Polycystic ovary syndrome (PCOS) is a common disorder affecting 4 to 21% reproductive-age women and also the leading cause of anovulatory infertility [1, 2]. Apart from its impact on ovulatory dysfunction, PCOS also affects overall health of women with long-lasting effects even in post-menstrual period [3]. Though first described in 1935, the etiology of the disease is still not well elucidated, which involves both genetic and environmental factors [4, 5]. Rotterdam criteria is the universally accepted diagnostic criteria and concludes the diagnosis of PCOS should be based on at least two of three major criteria, including oligo- or anovulation, clinical and/or biochemical signs of hyperandrogenism and polycystic ovaries identified by ultrasonography after excluding other androgen excess disorders [2]. Up to 80% affected women are overweight or obese, and insulin resistance (IR) occurs in most PCOS patients even in normal-weight population [6–8]. PCOS related obesity and IR also significantly increase the risk of diabetes mellitus, cardiovascular disease, and nonalcoholic steatohepatitis [9–11].

Glucagon-like peptide 1 is one of the most thoroughly studied incretins and its receptors are widely expressed in pancreas, gastrointestinal tract, heart, and even brain [12]. Agonists towards GLP1 receptors are recognized as popular anti-diabetic agents which can inhibit gastric emptying, increase measures of satiety, decrease food intake and thus cause weight reduction. GLP1RAs have recently become popular in diabetes and obese patients for its pronounced weight-losing effects, insulin-sensitizing function and additional cardiovascular protective benefits [13–16]. Furthermore, It has been discovered that GLP1R mRNA expressed in thalamus and hypothalamus, indicating potential ability of GLP1RAs to regulate GnRH from the hypothalamic neurons via a specific GLP-1R [17, 18]. Animal studies also have found GLP1 receptors expressed in ovary cells [19]. Improvement of reproductive dysfunction including follicles development, recovery of estrous and menstrual cycles and even reverse of polycystic ovary morphology were seen in several animal studies, though some studies presented with controversial results [20–24]. Given these, PCOS women particularly those obese population could benefit from GLP1RAs treatment.

A few clinical trials have shown that GLP1RAs administration improves pregnancy rate, menstrual frequency, obesity, excess of androgen and insulin levels in PCOS patients [25–28]. Former meta-analysis studies have mainly focused on anthropometric, metabolic, hormonal changes after GLP1RAs administration [29–31]. None has investigated conception rate changes or pregnancy outcomes which actually disturbed most patients. Herein, we perform this meta-analysis to evaluate the role of GLP1RAs in the management of PCOS patients especially with regard to reproductive health.

## Methods

This systematic review and meta-analysis was conducted according to the guidelines for the preferred reporting items for systematic reviews and meta-analyses (PRISMA) [32].

### Search strategy

A systematic search was performed in PubMed, Embase, Web of Science and Cochrane library covering the period up to September 22^nd^, 2022. The search terms used included the following: (“polycystic ovary syndrome” or “PCOS” or “polycystic ovary”) and (“Glucagon-Like Peptide 1” or “GLP1” or “liraglutide” or “exenatide”), and detailed search terms were given in Supplementary material. Additionally, manual checks of the reference lists within both the original literature and the reviews were also performed to identify eligible studies.

### Inclusion and exclusion criteria

Inclusion criteria were as follows: (1) Confirmed diagnosis of PCOS based on Rotterdam criteria, National Institute of Health criteria or Androgen Excess Society criteria, (2) GLP1RAs prescription as intervention, (3) Either a placebo or active agents as control, (4) reporting on pregnancy rate and/or menstrual regularity changes, (5) RCTs on human.

Exclusion criteria were as follows: (1) The diagnostic criteria were not clearly stated, (2) case reports, review articles, editorials, letters, conference abstracts, non-RCTs, etc., (3) studies without outcomes of interest, (4) studies without accessible data to perform quantitative analysis, (5) studies that were republished or duplicated using same participant cohorts. Articles in languages other than English were also excluded.

### Study selection and data extraction

LZ and HQ independently identified and selected relevant articles. Decisions on the inclusion of a full-text review were made and examined by both reviewers. Any discrepancies between the 2 investigators were solved by LY or discussion among all the reviewers.

Data were extracted from the studies including (1) study information: the trial name, author details, year of publication, country and diagnosis criteria for PCOS, (2) participant characteristics: age, baseline BMI, sample size and drugs(including dose usage and duration) of experimental and control groups, lifestyle instruction, (3) outcomes of interest: pregnancy rate; menstrual frequency changes; anthropometric parameters mainly BMI, waist circumference, body fat percent and HOMA-IR changes; alterations in SHBG level and androgen indexes including TT, fT, DHEAS and FAI. Similarly, the process were carried out by LZ and HQ separately. If discrepancies were found, the data in the original study were extracted again by discussion between the 2 reviewers until they reached an agreement.

### Quality assessment

The Cochrane risk of bias tool with RevMan 5.4 was utilized to assess the quality of the included studies [33]. Six domains of bias (selection, performance, detection, attrition, reporting, and other biases) were assessed. Two authors (LZ and HQ) independently assessed risk of bias in each study, using ‘low risk’, ‘high risk’ and ‘unclear risk’ of bias. Any disagreements were resolved by discussion, and the ultimate decision was reached with a third author.

### Statistical analysis

Statistical analysis was performed with Stata version 15.1 (Stata Corp., College Station, TX, USA) and RevMan 5.4 (The Nordic Cochrane Center, The Cochrane Collaboration). What we finally need is mean change and its corresponding standard deviation, which we can calculate based on baseline and after-treatment mean and SD [34]. One study presented with differences from baseline as mean (95% CI) [28], therefore we adopted an additional calculator tool available on Cochrane website to transform 95%CI into SD (available on: https://training.cochrane.org/resource/revman-calculator). Data of control groups were separated equally in two studies, as there were two experimental groups reported [25, 35]. Continuous data were expressed as WMD (same measure methods and units between groups) or SMD (different measure methods and units between groups), while dichotomous data were expressed as RR. All analyses were carried out using random-effects models for more conservative estimates. Heterogeneity between studies was evaluated by the *X*^2^ test and *I*^2^ statistic, and *P* values < 0.05 or *I*^2^ values > 50% were indicative of substantial heterogeneity. We also conducted subgroup analyses according to different drugs usage or other clinical variances such as race, medication duration etc. Furthermore, meta-regression analysis was performed to explore the potential factors for significant heterogeneity. A 2-tailed *P* value of less than 0.05 was considered statistically significant in all analyses. Sensitivity analyses were performed to examine the stability of pooled effect size results by serially excluding each study. Potential publication bias was evaluated by visual inspection of funnel plots as well as with Egger regression asymmetry test if the number of analyzed items was more than ten [36].

## Results

### Search results

**Fig. 1:**
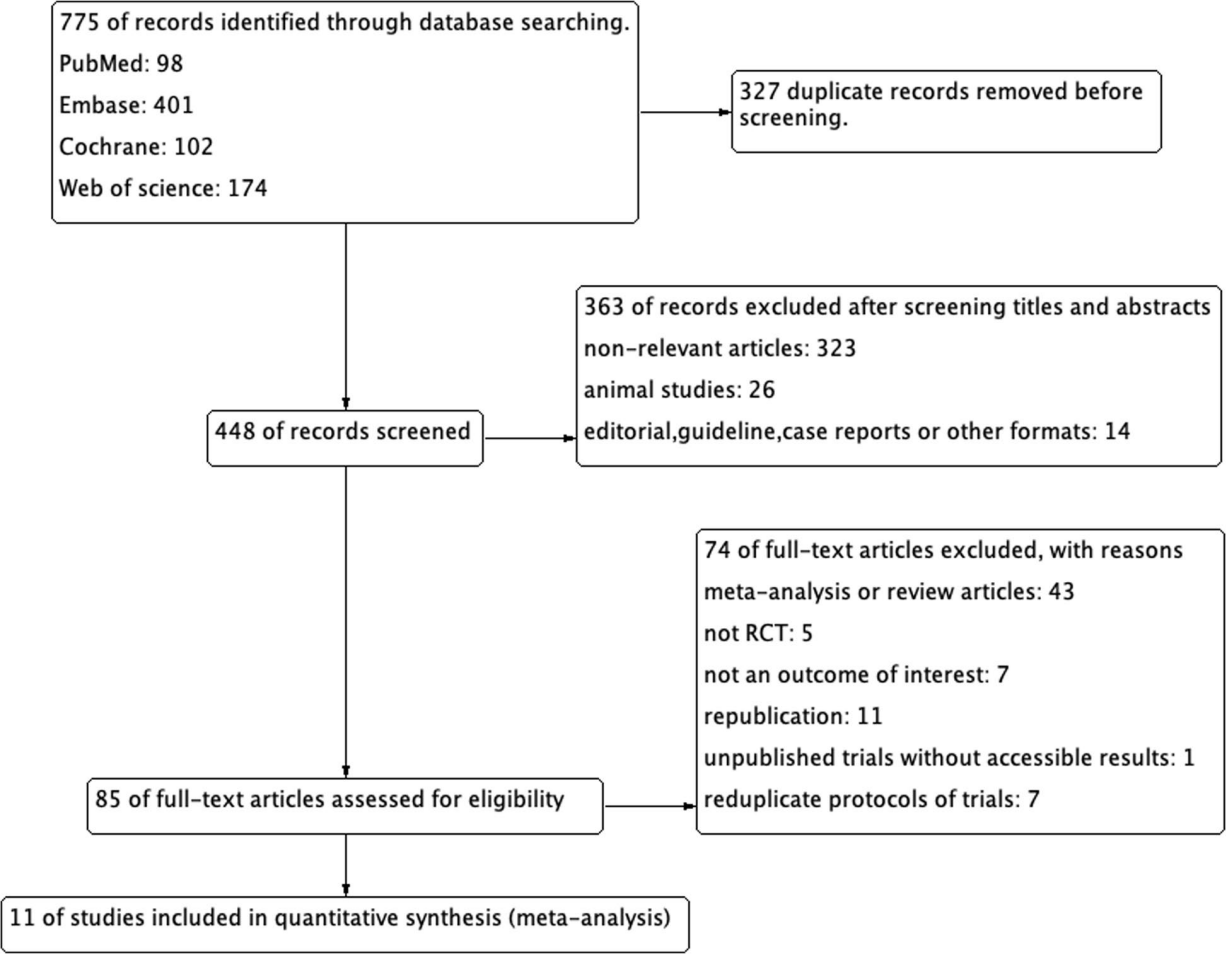
Flowchart showing the study selection process

As shown in Fig. 1, we identified 775 relevant studies of which 327 were duplicates, and 363 studies were excluded after screening titles and abstracts. The remaining 85 articles were further subjected to full-text review and 74 studies were subsequently excluded for reasons. Finally, a total of 11 studies were eligible for this meta-analysis [25–28, 35, 37–42].

### Basic characteristics

A pooled population of 840 patients was included, with 469 individuals in GLP1RAs arm and 371 individuals in control arm. Table 1 shows the characteristics of the included RCTs. Seven studies employed Rotterdam criteria for diagnosis and four used NIH criteria. All participants were aged 18 years older and were overweight or obese. Lifestyle modifications were promoted during medication in seven studies. Intervention groups received either GLP1RAs monotherapy or GLP1RAs plus metformin combined therapy, whilst patients in control groups used placebo or metformin alone. Exenatide was used in five studies [25–27, 38, 41] while liraglutide was adopted in others. GLP1RAs usage duration ranged from 12 to 32 weeks and dosage also varied from low dose (EXE 5ug bid) to maximum dose (LIRA 3 mg qd). In addition, since metformin is known to have beneficial effects in PCOS, any improvement in the prespecified outcome parameters or even equivalence would place GLP1RAs in a favorable position.

**Table 1:** Characteristics of the included RCTs

Authors (Publication year)	Diagnosis criteria (Country)	Interventionduration	Interventions (Sample size)	Lifestyle intervention	Trial group (Age;baseline BMI)	Control group (Age;baseline BMI)	Outcomes
Mojca Jensterle [37] (2015)	NICHD (Slovenia)	12wk	LIRA 1.2 mg QD (*n* = 17) vs MET 1000 mg BID (*n* = 15)	Not actively promoted	29.5 ± 7.7(y); 40.8 ± 6.1 kg/m^2^	25.3 ± 5.2(y); 38.2 ± 7 kg/m^2^	MF, weight, metabolic, endocrine changes, TBF
Karen Elkind-Hirsch [25] (2008)	Rotterdam (Louisiana)	24wk	COMBI (EXE 10 μg BID + MET 1000 mg BID) (*n* = 20) vs EXE 10 μg BID (*n* = 20) vs MET 1000 mg BID (*n* = 20)	On unrestricted diet	COMBI: 32.1 ± 0.7(y); 41.2 ± 1.7 kg/m^2^ EXE:28.2 ± 1.1(y); 39.9 ± 1.5 kg/m^2^	27.7 ± 1.3(y); 41.3 ± 1.8 kg/m^2^	MF, ovulation rate, insulin action, anthropometric measures, androgen levels, inflammatory markers
Renyuan Li [26] (2022)	Rotterdam (China)	12wk	EXE 5–10 μg BID (*n* = 80)*12wk + MET 1000 mg BID*52wk vs MET 1000 mg BID (*n* = 80)*12wk + MET 1000 mg BID*52wk	Actively promoted	28.19 ± 3.96(y); 29.07 ± 3.92 kg/m^2^	27.83 ± 3.52(y); 29.15 ± 4.11 kg/m^2^	Spontaneous and total PR, pregnancy outcomes, metabolic parameters
Juan Wang [27] (2017)	Rotterdam (China)	3 month	EXE 10 μg BID (*n* = 45) vs MET 500–2000 mg daily (*n* = 33)	NM	25.92 ± 6.75(y); 26.26 ± 5.71 kg/m^2^	25.67 ± 7.33(y); 25.74 ± 6.37 kg/m^2^	PR, sex hormone, ovulation rate, glucose metabolic index, AngII/Ang(1–7)
Malin Nylander [28] (2017)	Rotterdam (Denmark)	26wk	LIRA 1.8 mg QD (*n* = 48) vs Placebo 1.8 mg QD (*n* = 24)	NM	31.4(24.6–35.6)(y); 33.3 ± 5.1 kg/m^2^	26.2(24.8–31.5)(y); 33.3 ± 4.6 kg/m^2^	MF, ovarian and stromal volume, antral follicle count, sex hormone
Xin Liu [38] (2017)	Rotterdam (China)	12wk	EXE 10 μg BID*12wk + MET*12wk (*n* = 88) vs MET 1000 mg BID*12wk + MET*12wk (*n* = 88)	Promoted	27.93 ± 2.70(y); 29.16 ± 3.11 kg/m^2^	27.69 ± 3.80(y); 28.29 ± 1.86 kg/m^2^	Natural PR, MF, weight, BMI, TBF, metabolic and endocrine changes, inflammatory markers
Karen E. Elkind-Hirsch [39] (2022)	NIH (Louisiana)	32wk	LIRA 3 mg QD (*n* = 55) vs Placebo 3 mg QD (*n* = 27)	Promoted	31 ± 0.8(y); 41.6 ± 0.9 kg/m^2^	32 ± 1.1(y); 43.9 ± 1.5 kg/m^2^	MF, weight and FAI, waist circumference, BMI, sex hormone, OGTT result, insulin action, lipids profile, BP, TBF
Vesna Salamun [40] (2018)	Rotterdam (Slovenija)	12wk	COMBI (LIRA 1.2 mg QD + MET 1000 mg BID) (*n* = 14) vs MET 1000 mg BID (*n* = 14)	Actively promoted	30.1 ± 3.6(y); 37.8 ± 3.0 kg/m^2^	31.1 ± 4.7(y); 35.5 ± 4.9 kg/m^2^	Cumulative PR(IVF and spontaneous); oocyte and embryo quality, weight, metabolic and endocrine parameters, TBF
Siyuan Zheng [41] (2017)	Rotterdam (China)	12wk	EXE 10 μg BID (*n* = 41) vs MET 1000 mg BID (*n* = 41)	Promoted	27.7 ± 3.41(y); 29.18 ± 4.15 kg/m^2^	28.16 ± 3.92(y); 29 ± 4.1 kg/m^2^	MF, weight, hirsutism, metabolic and endocrine change, inflammatory marker levels
Mojca Jensterle [42] (2014)	NICHD (Slovenia)	12wk	LIRA 1.2 mg QD (*n* = 15) vs MET 1000 mg BID (*n* = 15)	Promoted	30.7 ± 7.9(y)^a^; 36.7 ± 5.6 kg/m^2^	30.7 ± 7.9(y)^a^; 39.4 ± 6.9 kg/m^2^	MF, weight, metabolic and endocrine change
Mojca Jensterle Sever [35] (2014)	NICHD (Slovenia)	12wk	COMBI (LIRA 1.2 mg QD + MET 1000 mg BID) (*n* = 13) vs LIRA 1.2 mg QD (*n* = 13) vs MET 1000 mg BID (*n* = 14)	Not actively promoted	COMBI:31.1 ± 5.1(y); 37.6 ± 5.1 kg/m^2^ LIRA:31.5 ± 6.4(y); 39.3 ± 4.2 kg/m^2^	31.3 ± 9.4(y); 36.6 ± 3.5 kg/m^2^	MF, weight, metabolic and endocrine change, TBF

^a^Age were not given separately

### Risk of bias of included studies

**Fig. 2:**
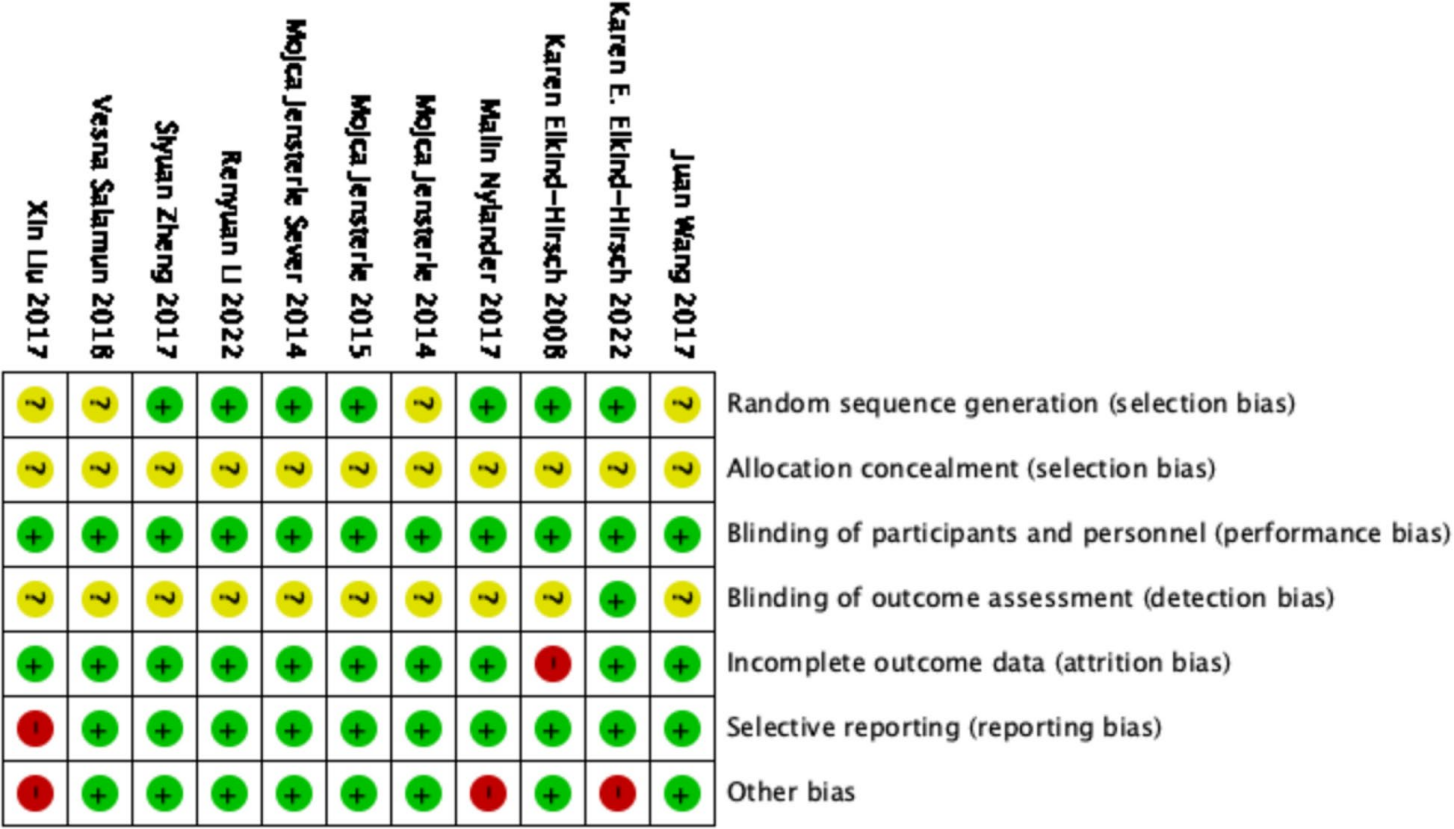
Assessment of the risk of bias

As shown in Fig. 2, adequate method of random sequence generation was described in seven trials, including RAND program in Excel or computer-generated randomization list [25, 26, 28, 35, 37, 39, 41]. The other four gave unspecific randomization procedure and had an unclear risk [27, 38, 40, 42]. None provided with a detail of allocation concealment, so it was considered an uncertain risk of selection bias. Two studies [28, 39] were double-blinded, one study [38] was single-blinded, other studies were open-label, but they were all considered to be at low risk of performance bias because outcome measures were objective results. Ten studies were assessed unclear risk of detection bias except study from Elkind-Hirsch et al. [39]. One study had a quite high missing rate (more than 30%) at final assessment, but information on the number of participants who dropped out and the corresponding reasons were given [25]. Study from Liu et al. was viewed of high risks in reporting bias and other bias [38, 43]. Others were at low risk for selecting outcome reports. Two studies received funding from pharmaceutical companies and thus were deemed to be at high risk for other bias [28, 39].

### Effect on pregnancy rate

**Fig. 3:**
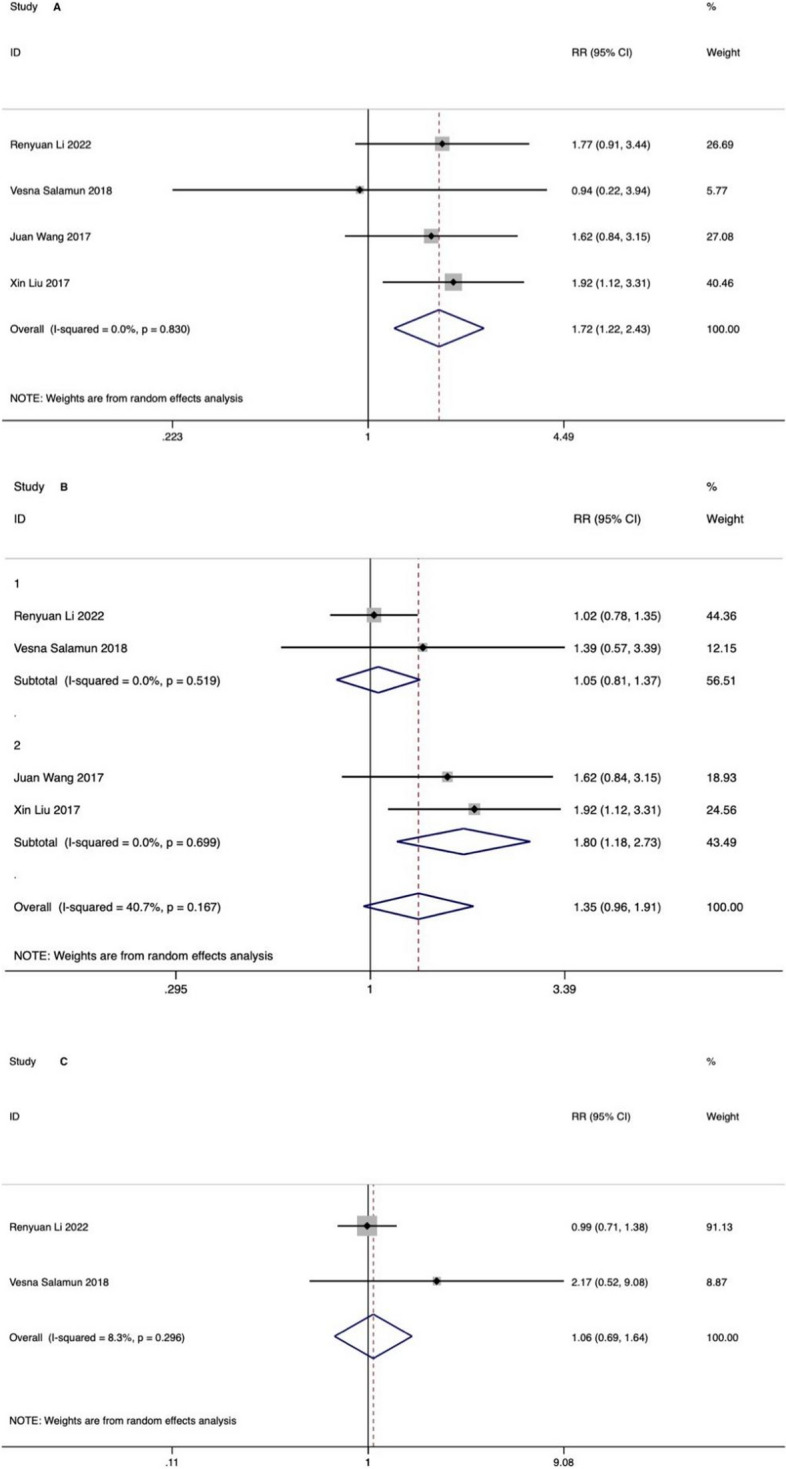
Effect on pregnancy rate: **A** natural pregnancy rate, **B** total pregnancy rate, **C** IVF pregnancy rate; 1 (follow-up more than 1 year), 2 (follow-up less than 1 year)

Four studies reported pregnancy rate in total. Significant improvements were observed in spontaneous PR (RR: 1.72, 95% CI 1.22 to 2.43, *I*^2^ = 0%, *P* = 0.002) without great heterogeneity. However, there was no difference in the IVF PR (RR: 1.06, 95% CI 0.69 to 1.64, *I*^2^ = 8.3%, *P* = 0.791) or total PR (RR: 1.35, 95% CI 0.96 to 1.91, *I*^2^ = 40.7%, *P* = 0.087). Subgroup analysis showed follow-up duration might explain the heterogeneity in total PR, that is, treatment with GLP1RAs could only improve total pregnancy rate in the following time period of less than 1 year (Fig. 3). Sensitivity analysis for total PR showed substantial change in heterogeneity when excluding Li et al.’s study [26], so as the estimate of effect size (RR 1.71; 95% CI 1.17 to 2.51, *I*^2^ = 0%, *P* = 0.005) (sensitivity analysis results shown in Supplementary Figs. S1-S3).

### Effect on menstrual cycles

**Fig. 4:**
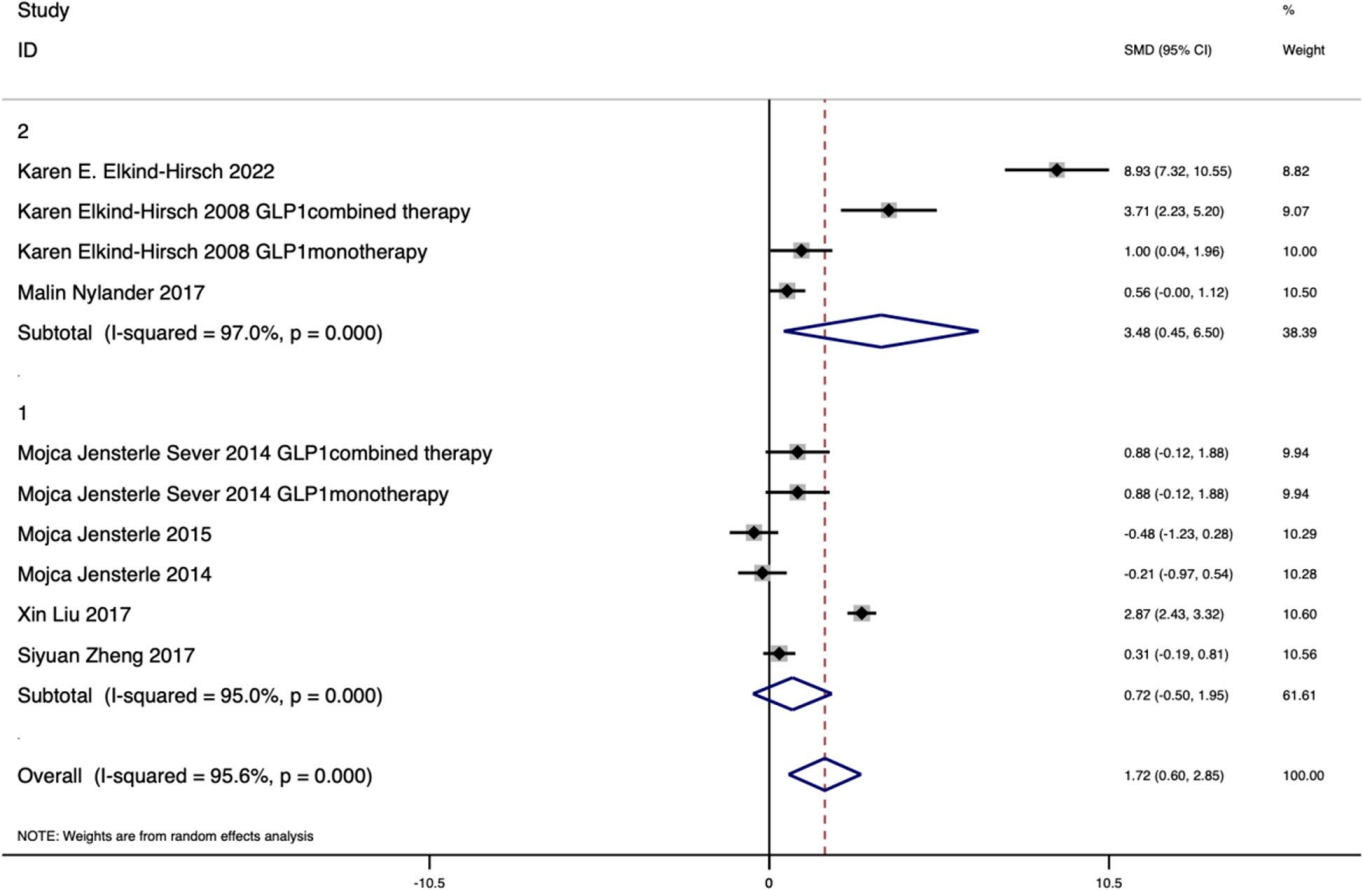
Effect on menstrual cycles: 1 (GLP1RAs treatment duration for 12wk), 2 (GLP1RAs treatment duration for more than 12wk)

Menstrual cyclicity was documented in eight RCTs. The pooled result revealed that adding GLP1RAs treatment was more effective than metformin alone or placebo in improving menstrual frequency (SMD: 1.72, 95% CI 0.60 to 2.85, *I*^2^ = 95.6%, *P* < 0.001). Subgroup analysis showed longer treatment duration was associated with menstrual cyclicity improvement, that is, PCOS participants who were treated with GLP1RAs for 24, 26 and 32 weeks respectively in three trials [25, 28, 39] had the most favourable changes in menstrual pattern compared those treated for 12 weeks (shown in Fig. 4). The heterogeneities were high in both overall analysis and subgroup analysis, but further sensitivity analysis demonstrated the result robust (shown in Supplementary Fig. S4). The meta-regression analysis also indicated treatment duration was a potential influencing factor (shown in Supplementary Tab. S1). The funnel plot for the menstrual patterns was given in Supplementary Fig. S5 (Harbord-Egger test, *P* = 0.55).

### Effect on anthropometrics and HOMA-IR

Significant improvements were observed in BMI and waist circumference, as presented in Table 2. Subgroup analysis indicated significant reduction in BMI were seen for GLP1RAs versus placebo or metformin, but not in combined treatment versus metformin monotherapy. There was no difference in total body fat percentage change in two arms and ethnicity might explain the heterogeneity through subgroup analysis. HOMA-IR was improved when adding GLP1RAs treatment. The funnel plots for BMI, WC and HOMA-IR were shown in Supplementary Figs. S7, S9 and S12 (Harbord-Egger test, *P* = 0.97, *P* = 0.67, *P* = 0.26, respectively) (sensitivity analysis results shown in Supplementary Figs. S6, S8, S10 and S11).

**Table 2:** Effect on anthropometrics and HOMA-IR

Study Group	No. of Studies/Subgroups	Heterogeneity	Effects Estimate (95% CI)	*P* value
BMI (WMD)	9	*P* = 0.147, *I*^*2*^ = 31.5%	-1.21 (-1.74, -0.68)	***P* < 0.001**
GLP1RA + MET vs MET	3	*P* = 0.825, *I*^*2*^ = 0%	-0.71 (-2.15, 0.73)	*P* = 0.335
GLP1RA vs MET	7	*P* = 0.218, *I*^*2*^ = 27.6%	-1.05 (-1.69, -0.42)	*P* = 0.001
GLP1RA vs Placebo	1	Not Applicable	-2.00 (-2.79, -1.21)	*P* < 0.001
WC (WMD)	9	*P* = 0.470, *I*^*2*^ = 0%	-2.90 (-3.65, -2.16)	***P* < 0.001**
TBF (WMD)	5	*P* = 0.003, *I*^*2*^ = 72.5%	-1.44 (-2.93, 0.05)	*P* = 0.058
European	5	*P* = 0.772, *I*^*2*^ = 0%	-1.84 (-4.60, 0.92)	*P* < 0.001
Chinese	1	Not Applicable	-1.30 (-1.73, -0.87)	*P* < 0.001
HOMA-IR (WMD)	10	*P* = 0.573, *I*^*2*^ = 0%	-0.65 (-0.80, -0.51)	***P* < 0.001**

### Effect on sex hormones

A slight reduction of TT was noticed in GLP1RAs group but the effect was gone after subgroup analysis and heterogeneity was still high, as demonstrated in Table 3. The pooled results showed no difference in fT or DHEAS levels but subgroup analysis showed a significant decrease in DHEAS when adding GLP1RAs to metformin monotherapy. GLP1RAs administration elevated SHBG compared to control group. A reduction of FAI was noticed in pooled result and subgroup analysis showed GLP1RAs did equally to metformin monotherapy. The funnel plots for TT and SHBG were shown in Supplementary Figs. S14 and S18 (Harbord-Egger test, *P* = 0.874, *P* = 0.214, respectively) (sensitivity analysis results shown in Supplementary Figs. S13-S19).

**Table 3:** Effect on sex hormones

Study Group	No. of Studies/subgroups	Heterogeneity	Effects Estimate(95% CI)	*P* Value
TT (SMD)	10	*P* < 0.001, *I*^*2*^ = 77.3%	-0.39 (-0.78, -0.002)	***P* = 0.049**
GLP1RA + MET vs MET	3	*P* = 0.008, *I*^*2*^ = 79.4%	-0.63 (-1.81, 0.55)	*P* = 0.293
GLP1RA vs MET	7	*P* = 0.009, *I*^*2*^ = 65%	-0.15 (-0.53, 0.24)	*P* = 0.465
GLP1RA vs Placebo	2	*P* = 0.001, *I*^*2*^ = 90.2%	-1.02 (-2.27, -0.23)	*P* = 0.110
fT(SMD)	5	*P* = 0.102, *I*^*2*^ = 45.5%	-0.16 (-0.59, 0.27)	*P* = 0.463
GLP1RA + MET vs MET	2	*P* = 0.671, *I*^*2*^ = 0%	-0.23 (-0.83, 0.36)	*P* = 0.444
GLP1RA vs MET	3	*P* = 0.109, *I*^*2*^ = 54.8%	0.16 (-0.57, 0.89)	*P* = 0.665
GLP1RA vs Placebo	1	Not Applicable	-0.66 (-1.19, -0.13)	*P* = 0.015
DHEAS (SMD)	7	*P* = 0.002, *I*^*2*^ = 67.1%	-0.40 (-0.82, 0.01)	*P* = 0.058
GLP1RA + MET vs MET	2	*P* = 0.375, *I*^*2*^ = 0%	-0.76 (-1.44, -0.08)	*P* = 0.029
GLP1RA vs MET	6	*P* = 0.557, *I*^*2*^ = 0%	-0.08 (-0.35, 0.18)	*P* = 0.542
GLP1RA vs Placebo	1	Not Applicable	-1.43 (-1.99, -0.87)	*P* < 0.001
SHBG (WMD)	9	*P* = 0.099, *I*^*2*^ = 37.5%	4.42 (2.18, 6.67)	***P* < 0.001**
GLP1RA + MET vs MET	3	*P* = 0.665, *I*^*2*^ = 0%	10.19 (6.29, 14.09)	*P* < 0.001
GLP1RA vs MET	7	*P* = 0.203, *I*^*2*^ = 29.5%	3.07 (0.63, 5.51)	*P* = 0.014
GLP1RA vs Placebo	1	Not Applicable	5.40 (-0.25, 11.05)	*P* = 0.061
FAI(WMD)	7	*P* = 0.019, *I*^*2*^ = 58.4%	-1.55 (-2.59, -0.51)	***P* = 0.004**
GLP1RA + MET vs MET	1	Not Applicable	-3.50 (-5.39, -1.61)	*P* < 0.001
GLP1RA vs MET	5	*P* = 0.037, *I*^*2*^ = 60.8%	-0.04 (-2.41, 2.32)	*P* = 0.973
GLP1RA vs Placebo	2	*P* = 0.570, *I*^*2*^ = 0%	-1.74 (-2.05, -1.43)	*P* < 0.001

## Discussion

To our best knowledge, it is the first meta-analysis exploring the role of GLP1RAs in reproductive aspects of PCOS women. Our study indicates a significant improvement in natural pregnancy rate following GLP1RAs intervention, and total pregnancy rate also elevates though merely within a short period. However, there is no increase in the rate of IVF pregnancy. Longer duration of GLP1RAs prescription leads to a more favorable menstrual regularity. Besides, GLP1RAs treatment is associated with improvement in HOMA-IR, increment in SHBG, reduction in BMI, WC and TT but not in fT, DHEAS or FAI. A decrease in TBF is also noticed in European population subgroup.

Main alterations in HPO axis in PCOS contain an increase of LH activity, follicles resistance to FSH, hypersecretion of androgens from ovarian theca cell and thereby inhibition of dominant follicle maturation and regular ovulating. The prevalence of infertility and menstrual disorders among PCOS women ranges from approximately 40% to 75%, and almost 40% PCOS patients fail to conceive spontaneously and turn to seek help from ART [1, 2, 44]. Besides, they have a higher chance encountering complications during pregnancy or even beyond, including miscarriage, GDM, preeclampsia, premature delivery, increased perinatal mortality and a higher risk of metabolic disorders for offspring [45, 46].

Here in this study, natural pregnancy rate with GLP1RAs prescription alone or in combination with metformin was improved when compared to metformin monotherapy. And the pooled effect size was not reversed by individual studies, indicating the result robust. Given that the estimated RR was increased after exclusion of the study by Wang J et al. [27] wherein metformin was not provided in the GLP1RAs group, we assume that the combination therapy of GLP1RAs plus metformin may be more efficient than either GLP1RAs or metformin monotherapy in terms of raising natural pregnancy rate. A similar result was not found in IVF pregnancy rate, which may be partly because it also relies on the clinical experience of fertility doctors and techniques of IVF procedures. Subgroup analysis result showed that total pregnancy rate elevated during a temporary period but not in the following 1-year-long time after such a short term usage (no more than 12 weeks) of GLP1RAs. It is also worth exploring whether a extension of GLP1RAs treatment duration could impose a more long-lasting improvement in pregnancy rate among PCOS patients.

To be mentioned with, baseline characteristics and intervention in four studies focusing on pregnancy rate were quite different, though without great heterogeneity. Firstly, participants in study from Salamun et al. were poorly responsive to lifestyle modifications and resistant to the first-line reproductive treatments with CC or aromatase inhibitors previously, which might underestimate pregnancy rate after GLP1RAs. Secondly, though treatment duration of GLP1RAs were similar, the drug species, dosage, concomitant medication (metformin) usage and its corresponding continuance varied: one study compared GLP1RAs monotherapy with metformin [27], however the others also employed metformin in experimental groups which may exert an add-on effect. Thirdly, Salamun et al. adopted a drug washout period after interventions, Wang et al. combined with CC in both groups while all patients in Liu and Li et al.’s study were switched to metformin treatment for another 12 weeks and 52 weeks respectively until pregnancy confirmed. Lastly, we intended to include RCTs to reduce the likelihood of recall and selection bias, but among four studies, three of them were open-label and one was single-blind clinical trials, which to some extent increased the risk of performance and detection bias.

Menstrual frequency also increased in 273 participants treated with GLP1RAs versus 208 patients in control arms, though the heterogeneity was quite high due to different intervention protocols and the enrolling of PCOS patients of different phenotypes. Further meta regression analysis showed treatment duration might be the source of heterogeneity. Those who received 12 weeks of GLP1RAs therapy had an increase in menstrual frequency but not in a statistically significant manner. Meanwhile, patients in the longer-duration subgroup had a more preferable improvement. Accordingly, we recommend that a continuous administration of GLP1RAs for at least 24 weeks may be plausible to restore a regular menstrual cycle [25]. However, due to the high heterogeneity, inferences should be drawn cautiously and further large-scale, well-designed RCTs are warranted to determine a minimum treatment duration.

Significant enhancements in anthropometric parameters and insulin sensitivity were obtained in GLP1RAs group, which were also in line with former studies [30, 31]. Waist girth, a simple reflection of abdominal obesity, decreased profoundly but the reduction of total body fat was simply seen in European patients. The improvement in SHBG level may be attributed to increased insulin sensitivity. GLP1RAs monotherapy did equally to metformin in androgen excess, but there seemed to be an add-on effect of GLP1RAs in lowering DHEAS and FAI. Further studies can be carried out to discuss the effect of GLP1RAs on body composition, fat distribution change, hyperandrogenism in people from different ethnic backgrounds.

Of note, GLP1RAs is a category C drug for pregnancy by FDA and EMA and its administration in women preparing for pregnancy should include a washout period. There are evidences of fetus toxicity in animal studies, but a few studies have reported a normal birth and no adverse outcomes in women exposed to GLP1RAs in their first terms of pregnancy [47, 48]. Only one trial herein documented the incidence rate of gestational complications and adverse outcomes, which turned out to be similar between groups [26]. Due to the scarce of the human studies investigating the safety of GLP1RAs usage before or during gestation, the related meta-analysis result cannot be performed here.

The plausible mechanisms underlying the beneficial effect of GLP1RAs on the reproductive function in PCOS women are listed as below. Firstly, obesity and insulin resistance, as aggravating factors for infertility, are alleviated within GLP1RAs usage. It is suggested that an increase in pregnancy rate is in accordance with a reduction in HOMA-IR [49]. Higher circulating insulin level is associated with subsequent excess ovarian androgen production and reduction of SHBG. A negative feedback on HPO axis from androgens aromatization in adipose tissues worsens gonadotropin activity. GLP1RAs administration demonstrates substantial insulin sensitivity enhancement and profound weight loss effect and thus inhibits a vicious cycle among obesity, IR, hyperandrogenism, which promise its benefits in PCOS patients [15, 20, 22, 50].

Moreover, as aforementioned, GLP-1 receptors are widely distributed, including HPO axis. Preclinical researches have provided an additional perspective that GLP1RAs exerts effects directly via hypothalamus-pituitary-gonadal axis. For instance, GLP-1 and Exendin-4 can act on the gonadal axis, involving the hypothalamic kiss-1 system, to influence reproductive efficiency in female rats [51]. Wu et al. demonstrated that dulaglutide, a long-acting GLP1RA, may reduce the hyperandrogenemia of PCOS rats by regulating the expression of steroid hormone synthesis related gene proteins in the ovary and thus improving the morphology of their polycystic ovaries [52]. Granulosa cells in ovaries are essential in the follicular development and dominant follicle selection. Sun et al. reported the contribution of GLP-1 to the regulation of ovarian granulosa cells proliferation and antiapoptosis, thereby promoting oocyte maturation in PCOS rats [53]. To sum up, preclinical studies have found potential direct roles of GLP1RAs in HPO axis, but whether the conclusion could be extended to human beings needs to be verified.

In addition, low-grade chronic inflammation and adipose tissue dysfunction cannot be ignored. Higher levels of inflammatory markers including CRP, IL-18, IL-6 and TNF-α in PCOS women have been widely reported [54, 55]. Artunc-Ulkumen et al. found exenatide could protect endometrial and ovarian microenvironments against oxidative stress, fibrosis, and degeneration [56]. Previous studies also have showed a decrease in inflammatory markers in PCOS patients treated with GLP1RAs, suggesting an anti-inflammatory effect of GLP1RAs at the cellular and molecular level [21, 57].

### Strengths and limitations

This meta-analysis incorporated recently published studies and addressed the potential role of GLP1RAs in reproductive aspects in PCOS patients. Since no prior meta-analysis has focused on the improvement of pregnancy rate, menstrual frequency, hyperandrogenism, HOMA-IR, and obesity-related parameters simultaneously, our study is more complete and thorough. Besides, we also performed a subgroup analysis to investigate the effects of GLP1RAs monotherapy or combination with metformin in the management of PCOS. Meanwhile, we utilized subgroup analysis, meta-regression, and sensitivity analysis to explain heterogeneities in pooled results.

However, our acceptances to both experimental and control group due to the paucity of available data, a lack of information on which disease phenotype of each participants belonged to and no uniformity of lifestyle intervention, GLP1RAs type or treatment duration, led to a moderate to high heterogeneity in some main outcomes. Secondly, participants from some studies also received metformin treatment, the results should be interpreted with caution since a true comparison between GLP1RAs and placebo was not available. Furthermore, most studies enrolled obese participants and were not specifically designed to evaluate menstrual patterns, which might cause bias. Finally, the small number of studies in subgroup and meta-regression analysis may have allowed for under powered analyses results.

## Conclusions

The present study reveals that the use of GLP-1RAs contributes to a higher natural pregnancy rate and a more regular menstrual frequency, improvement in obesity, insulin resistance, gonadal parameters mainly on SHBG. It is worth noting that GLP1RAs treatment in obese PCOS patients can be a new therapeutic option beyond the goal of weight loss. Still, more long-term, large-scale, multi-ethnic, phenotype-specific, well-designed trials are warranted to confirm the efficacy and safety of GLP1RAs in preconceptional PCOS women. Besides, intensive research into the mechanisms by which GLP1RAs affect the reproductive health is still needed.

### Supplementary Information

**Additional file 1: Fig. S1.** Sensitivity analysis of natural pregnancy rate. **Fig. S2.** Sensitivity analysis of total pregnancy rate. **Fig. S3.** Sensitivity analysis of IVF pregnancy rate. **Fig. S4.** Sensitivity analysis of menstrual cycles. **Fig. S5.** Funnel plot of menstrual cycles. **Tab. S1.** Summary of Meta-Regression Analyses for Menstrual Cycles. **Fig. S6.** Sensitivity analysis of BMI. **Fig. S7.** Funnel plot of BMI. **Fig. S8.** Sensitivity analysis of WC. **Fig. S9.** Funnel plot of WC. **Fig. S10.** Sensitivity analysis of TBF. **Fig. S11.** Sensitivity analysis of HOMA-IR. **Fig. S12.** Funnel plot of HOMA-IR. **Fig. S13.** Sensitivity analysis of TT. **Fig. S14.** Funnel plot of TT. **Fig. S15.** Sensitivity analysis of fT. **Fig. S16.** Sensitivity analysis of DHEAS. **Fig. S17.** Sensitivity analysis of SHBG. **Fig. S18.** Funnel plot of SHBG. **Fig. S19.** Sensitivity analysis of FAI.

## Data Availability

The original contributions presented in the study are included in the article or supplementary material. Further inquiries can be directed to the corresponding author.

## Abbreviations

GLP1RAs: Glucagon-like peptide 1 receptor agonists
PCOS: Polycystic ovary syndrome
RCT: Randomized controlled trial
WMD: Weighted mean difference
SMD: Standardized mean difference
RR: Risks ratio
CI: Confidence interval
SD: Standard deviations
IR: Insulin resistance
GI: Gastrointestinal
GnRH: Gonadotropin-releasing hormone
LIRA: Liraglutuide
EXE: Exenatide
MET: Metformin
CC: Clomifene citrate
NM: Not mentioned
BID: Twice a day
QD: Once a day
COMBI: Combined group
MF: Menstrual frequency
PR: Pregnancy rate
IVF: In vitro fertilization
TBF: Total body fat
BMI: Body mass index
WC: Waist circumference
HOMA-IR: Homeostasis model assessment-insulin resistance
TT: Total testosterone
fT: Free testosterone
FAI: Free androgen index
DHEAS: Dehydroepiandrosterone sulphate
SHBG: Sex hormone binding globulin
OGTT: Oral glucose tolerance test
BP: Blood pressure
ART: Assisted reproductive technology
GDM: Gestational diabetes mellitus
FDA: Food and Drug Administration
EMA: European Medicines Agency
HPO axis: Hypothalamus pituitary ovary axis
CRP: C-reactive protein
IL-18: Interleukin 18
TNF-α: Tumor necrosis factor α
IL-6: Interleukin 6
