# Supplementary material for: Effects of GLP1RAs on pregnancy rate and menstrual cyclicity in women with polycystic ovary syndrome: a meta-analysis and systematic review
Source: BMC Endocr Disord. 2023 Nov 8;23:245. doi: 10.1186/s12902-023-01500-5 (PMC10631119; doi:10.1186/s12902-023-01500-5)
Supplement: Supplementary file 1 — Additional file 1: Fig. S1. Sensitivity analysis of natural pregnancy rate. Fig. S2. Sensitivity analysis of total pregnancy rate. Fig. S3. Sensitivity analysis of IVF pregnancy rate. Fig. S4. Sensitivity analysis of menstrual cycles. Fig. S5. Funnel plot of menstrual cycles. Tab. S1. Summary of Meta-Regression Analyses for Menstrual Cycles. Fig. S6. Sensitivity analysis of BMI. Fig. S7. Funnel plot of BMI. Fig. S8. Sensitivity analysis of WC. Fig. S9. Funnel plot of WC. Fig. S10. Sensitivity analysis of TBF. Fig. S11. Sensitivity analysis of HOMA-IR. Fig. S12. Funnel plot of HOMA-IR. Fig. S13. Sensitivity analysis of TT. Fig. S14. Funnel plot of TT. Fig. S15. Sensitivity analysis of fT. Fig. S16. Sensitivity analysis of DHEAS. Fig. S17. Sensitivity analysis of SHBG. Fig. S18. Funnel plot of SHBG. Fig. S19. Sensitivity analysis of FAI. [file 12902_2023_1500_MOESM1_ESM.docx]

**Search strategies**

**Pubmed:**

("glucagon like peptide 1"[MeSH Terms] OR "glucagon like peptide 1"[Title/Abstract] OR "GLP-1"[Title/Abstract] OR "GLP-1"[Title/Abstract] OR "glucagon like peptide 1"[Title/Abstract] OR "Liraglutide"[Title/Abstract] OR "Semaglutide"[Title/Abstract] OR "Dulaglutide"[Title/Abstract] OR "Exenatide"[Title/Abstract] OR "Lixisenatide"[Title/Abstract] OR "Loxenatide"[Title/Abstract] OR "Albiglutide"[Title/Abstract]) AND ("polycystic ovary syndrome"[MeSH Terms] OR "ovary syndrome polycystic"[Title/Abstract] OR "syndrome polycystic ovary"[Title/Abstract] OR "stein leventhal syndrome"[Title/Abstract] OR "stein leventhal syndrome"[Title/Abstract] OR "syndrome stein leventhal"[Title/Abstract] OR "sclerocystic ovarian degeneration"[Title/Abstract] OR ((("Ovarian"[All Fields] OR "ovarians"[All Fields]) AND ("degenerate"[All Fields] OR "degenerated"[All Fields] OR "degenerately"[All Fields] OR "degenerates"[All Fields] OR "degenerating"[All Fields] OR "Degeneration"[All Fields] OR "degenerations"[All Fields])) AND "Sclerocystic"[Title/Abstract]) OR "sclerocystic ovary syndrome"[Title/Abstract] OR "polycystic ovarian syndrome"[Title/Abstract] OR "ovarian syndrome polycystic"[Title/Abstract] OR "polycystic ovary syndrome 1"[Title/Abstract] OR "sclerocystic ovaries"[Title/Abstract] OR (("ovarial"[All Fields] OR "Ovary"[MeSH Terms] OR "Ovary"[All Fields] OR "Ovaries"[All Fields] OR "ovary s"[All Fields]) AND "Sclerocystic"[Title/Abstract]) OR "sclerocystic ovary"[Title/Abstract])

**Embase:**

('ovary polycystic disease'/exp OR 'stein leventhal syndrome' OR 'cystic ovary' OR 'micropolycystic ovary' OR 'multiple follicle cyst' OR 'ovary polycystic disease' OR 'ovary polycystic syndrome' OR 'ovary, micropolycystic' OR 'ovary, polycystic' OR 'polycystic ovarian disease' OR 'polycystic ovary' OR 'polycystic ovary disease' OR 'polycystic ovary syndrome' OR 'stein cohen leventhal syndrome' OR 'stein leventhal disease' OR 'syndrome stein leventhal') AND ('glucagon like peptide 1 receptor agonist'/exp OR 'glp 1 agonist' OR 'glp 1 receptor agonist' OR 'glucagon like peptide 1 agonist' OR 'glucagon like peptide 1 receptor agonist' OR 'glucagon like peptide 1 receptor stimulating agent' OR 'long acting glp 1 agonist' OR 'long acting glp 1 receptor agonist' OR 'long acting glucagon like peptide 1 agonist' OR 'long acting glucagon like peptide 1 receptor agonist' OR liraglutide OR semaglutide OR dulaglutide OR exenatide OR lixisenatide OR loxenatide OR albiglutide)

**Web of Science:**

(TS=(Glucagon-Like Peptide 1) OR AB=( GLP-1 OR GLP1 OR Glucagon-Like Peptide-1 OR Liraglutide OR Semaglutide OR Dulaglutide OR Exenatide OR Lixisenatide OR Loxenatide OR Albiglutide)) AND (TS=(Polycystic Ovary Syndrome) OR AB=(Ovary Syndrome, Polycystic OR Syndrome, Polycystic Ovary OR Stein-Leventhal Syndrome OR Stein Leventhal Syndrome OR Syndrome, Stein-Leventhal OR Sclerocystic Ovarian Degeneration OR Ovarian Degeneration, Sclerocystic OR Sclerocystic Ovary Syndrome OR Polycystic Ovarian Syndrome OR Ovarian Syndrome, Polycystic OR Polycystic Ovary Syndrome 1 OR Sclerocystic Ovaries OR Ovary, Sclerocystic OR Sclerocystic Ovary))

**Cochrane:**

#1 MeSH descriptor: [Glucagon-Like Peptide 1] explode all trees 1970

#2 (GLP-1) 3911

#3 (GLP 1) 4294

#4 (Glucagon Like Peptide 1) 4148

#5 (Glucagon-Like Peptide-1) 3887

#6 Liraglutide 2170

#7 Semaglutide 749

#8 Dulaglutide 470

#9 Exenatide 1298

#10 Lixisenatide 345

#11 Loxenatide 15

#12 Albiglutide 145

#13 #1 OR #2 OR #3 OR #4 OR #5 OR #6 OR #7 OR #8 OR #9 OR #10 OR #11 OR #12 8113

#14 MeSH descriptor: [Polycystic Ovary Syndrome] explode all trees 1726

#15 (Ovary Syndrome, Polycystic) 3983

#16 (Syndrome, Polycystic Ovary) 3983

#17 (Stein-Leventhal Syndrome) 54

#18 (Stein Leventhal Syndrome) 56

#19 (Syndrome, Stein-Leventhal) 54

#20 (Sclerocystic Ovarian Degeneration) 5

#21 (Ovarian Degeneration, Sclerocystic) 5

#22 (Sclerocystic Ovary Syndrome) 44

#23 (Polycystic Ovarian Syndrome) 2093

#24 (Ovarian Syndrome, Polycystic) 2093

#25 (Polycystic Ovary Syndrome 1) 2692

#26 (Sclerocystic Ovaries) 15

#27 (Ovary, Sclerocystic) 44

#28 (Sclerocystic Ovary) 44

#29 #14 OR #15 OR #16 OR #17 OR #18 OR #19 OR #20 OR #21 OR #22 OR #23 OR #24 OR #25 OR #26 OR #27 OR #28 4413

#30 #13 AND #29 102


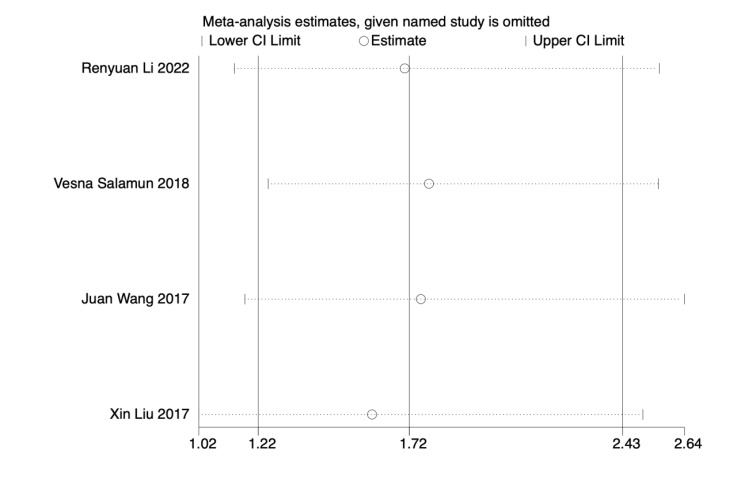


**Fig. S1** Sensitivity analysis of natural pregnancy rate
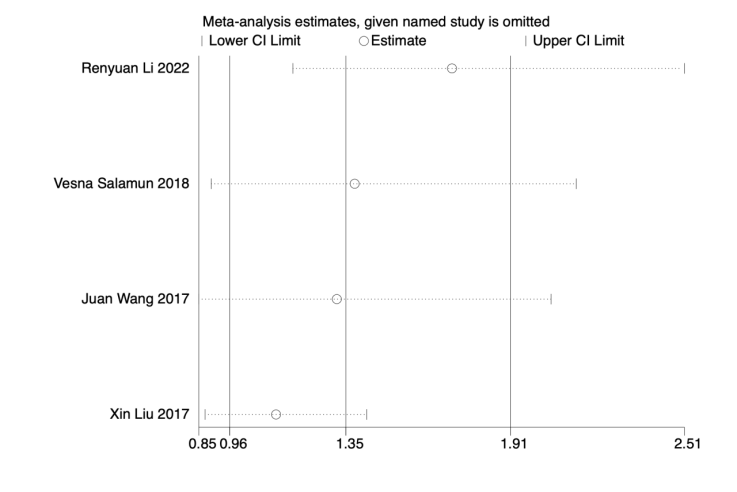
**Fig. S2** Sensitivity analysis of total pregnancy rate


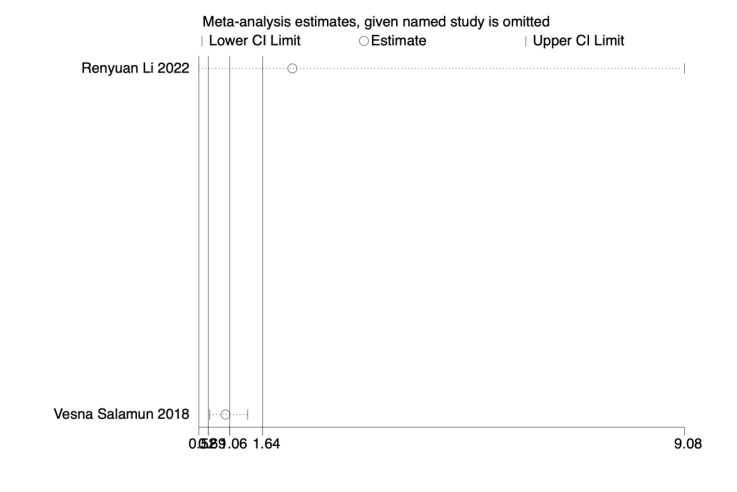


**Fig. S3** Sensitivity analysis of IVF pregnancy rate


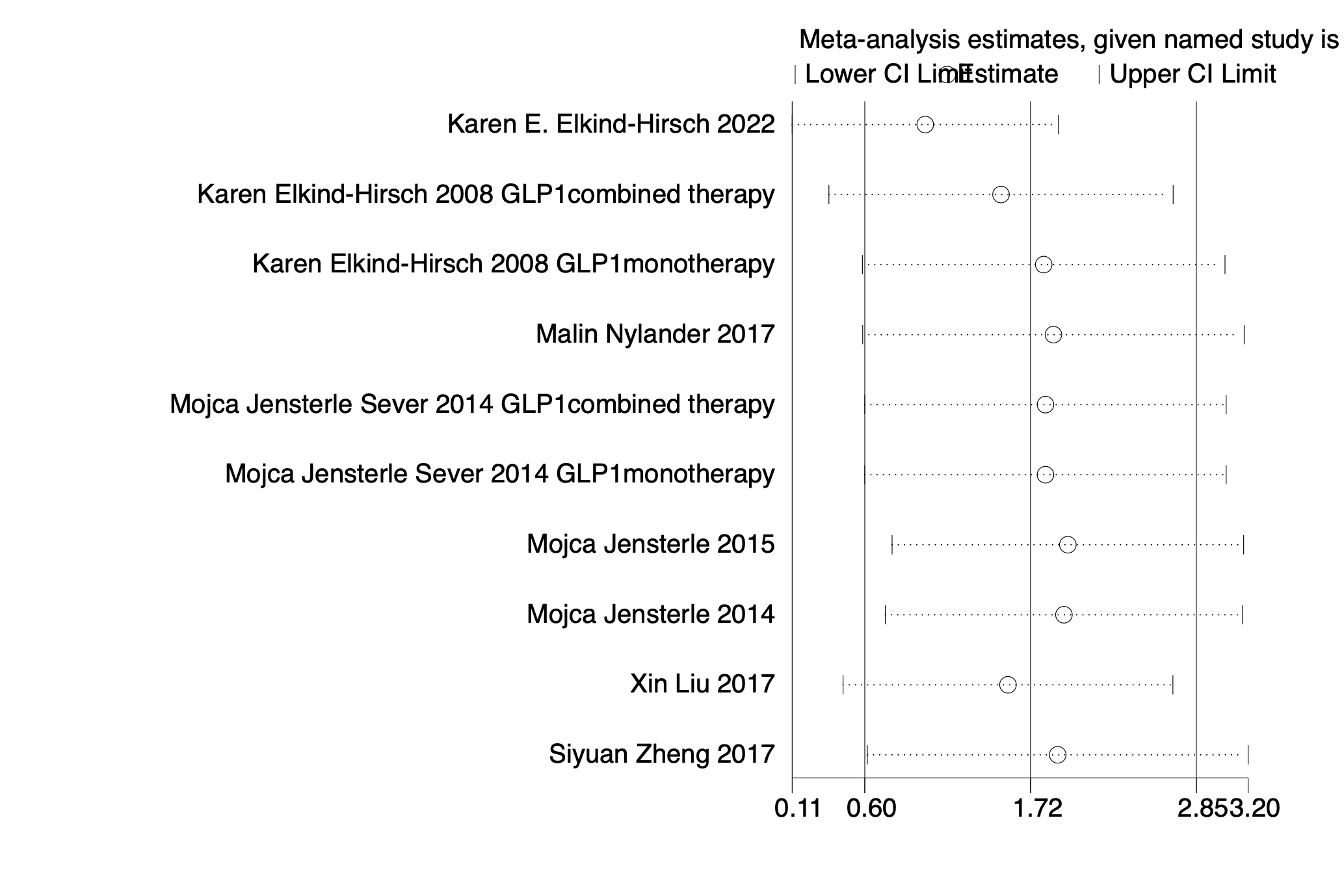
**Fig. S4** Sensitivity analysis of menstrual cycles


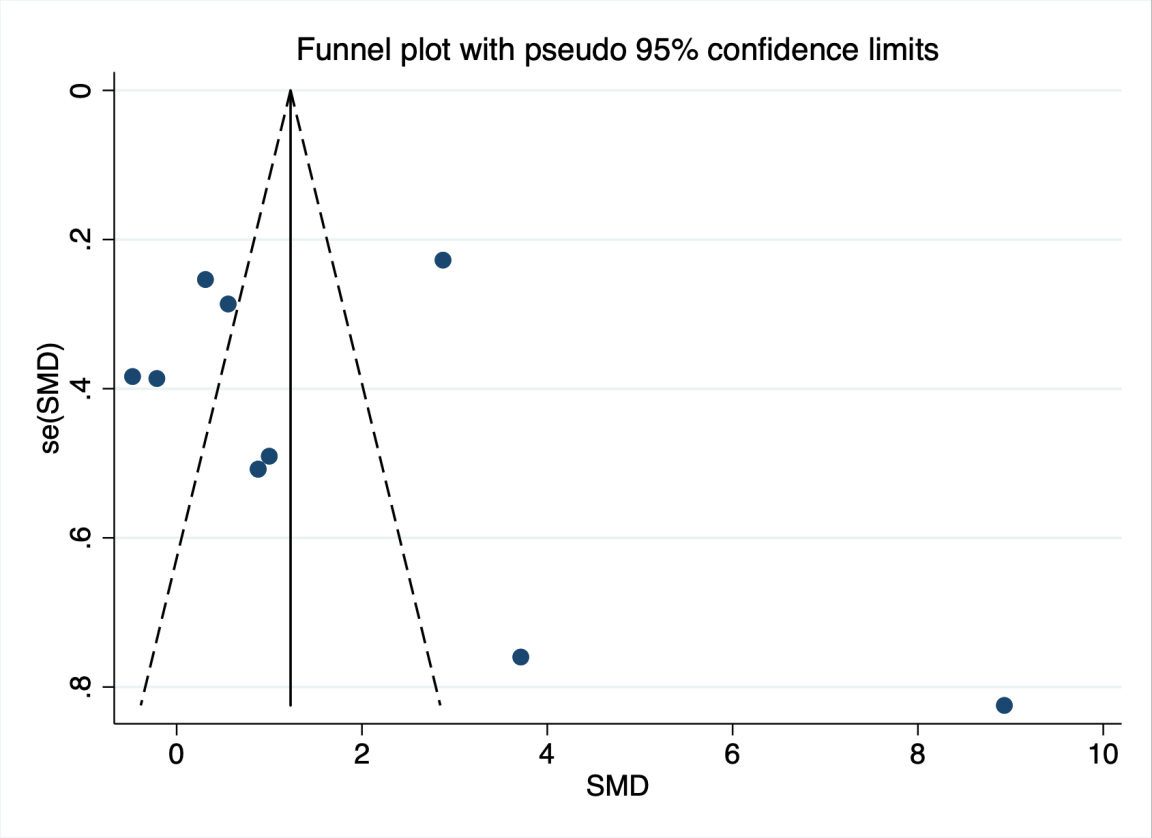


**Fig. S5** Funnel plot of menstrual cycles

**Tab. S1** Summary of Meta-Regression Analyses for Menstrual Cycles

| Outcome | Factor | Coefficient(β) | CI | SE | *P* value |
| --- | --- | --- | --- | --- | --- |
| Menstrual cyclicity | Ethnicity as Chinese | 9.1743 | -1.5476 to 19.8962 | 3.3691 | 0.072 |
|  | Diagnosis with Rotterdam Criteria | -7.8042 | -15.7723 to 0.1640 | 2.5038 | 0.053 |
|  | Lifestyle Modification | 0.5720 | -4.2480 to 5.3920 | 1.5146 | 0.731 |
|  | GLP1RAs using more than 12wk | 9.7946 | 2.0570 to 17.5322 | 2.4313 | **0.027** |
|  | GLP1RAs-MET Combined treatment | 1.3043 | -3.1249 to 5.7336 | 1.3918 | 0.418 |
|  | MET as control | 1.0896 | -4.5101 to 6.6893 | 1.7595 | 0.580 |


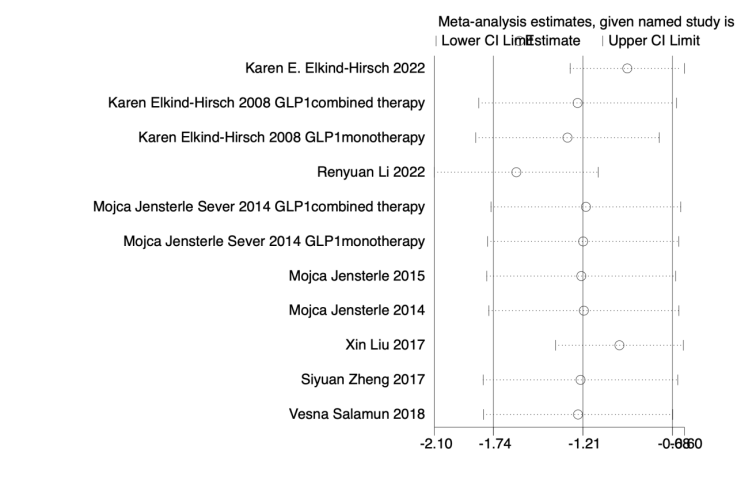


**Fig. S6** Sensitivity analysis of BMI


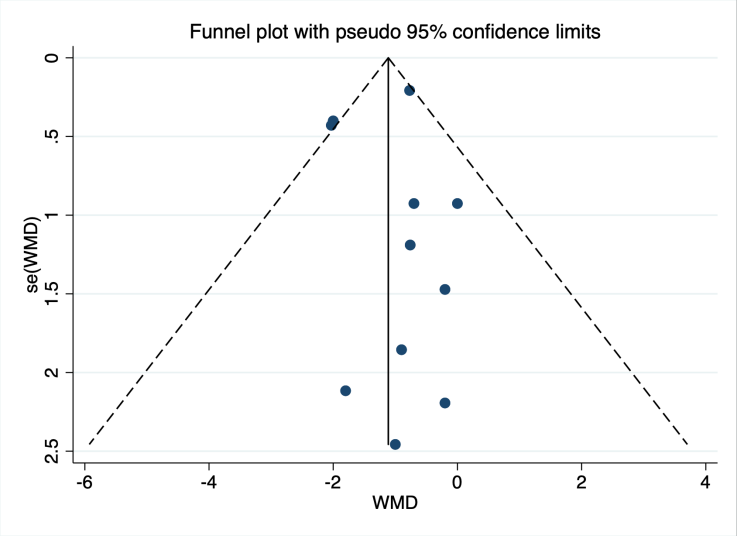


**Fig. S7** Funnel plot of BMI


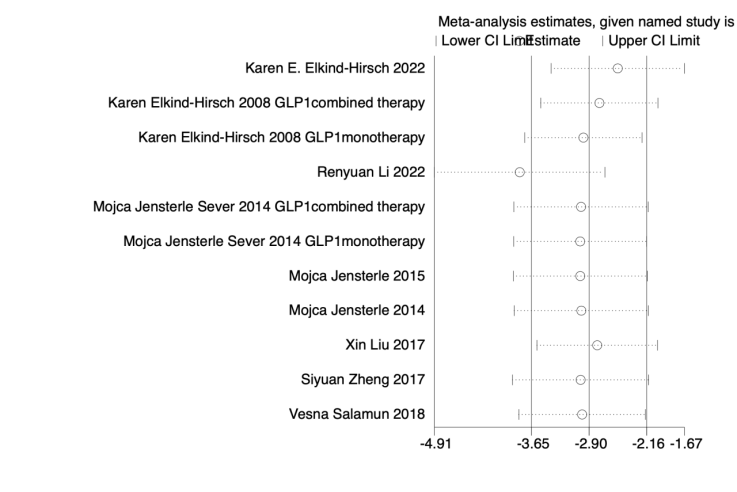


**Fig. S8** Sensitivity analysis of WC


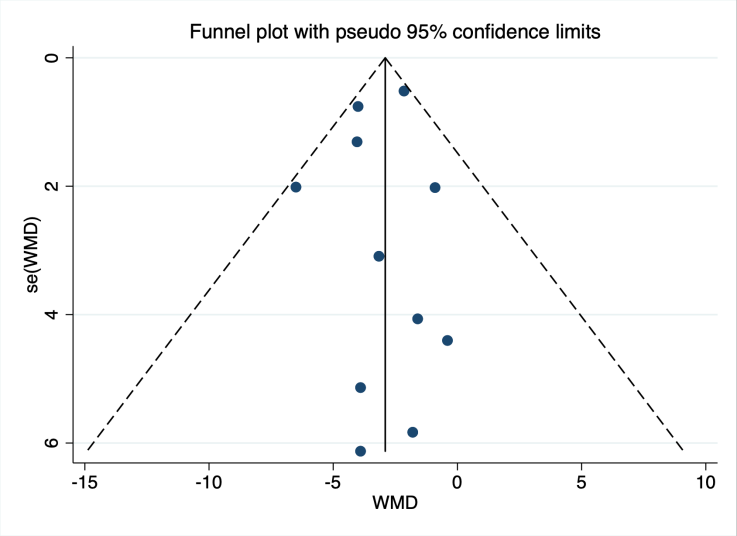


**Fig. S9** Funnel plot of WC


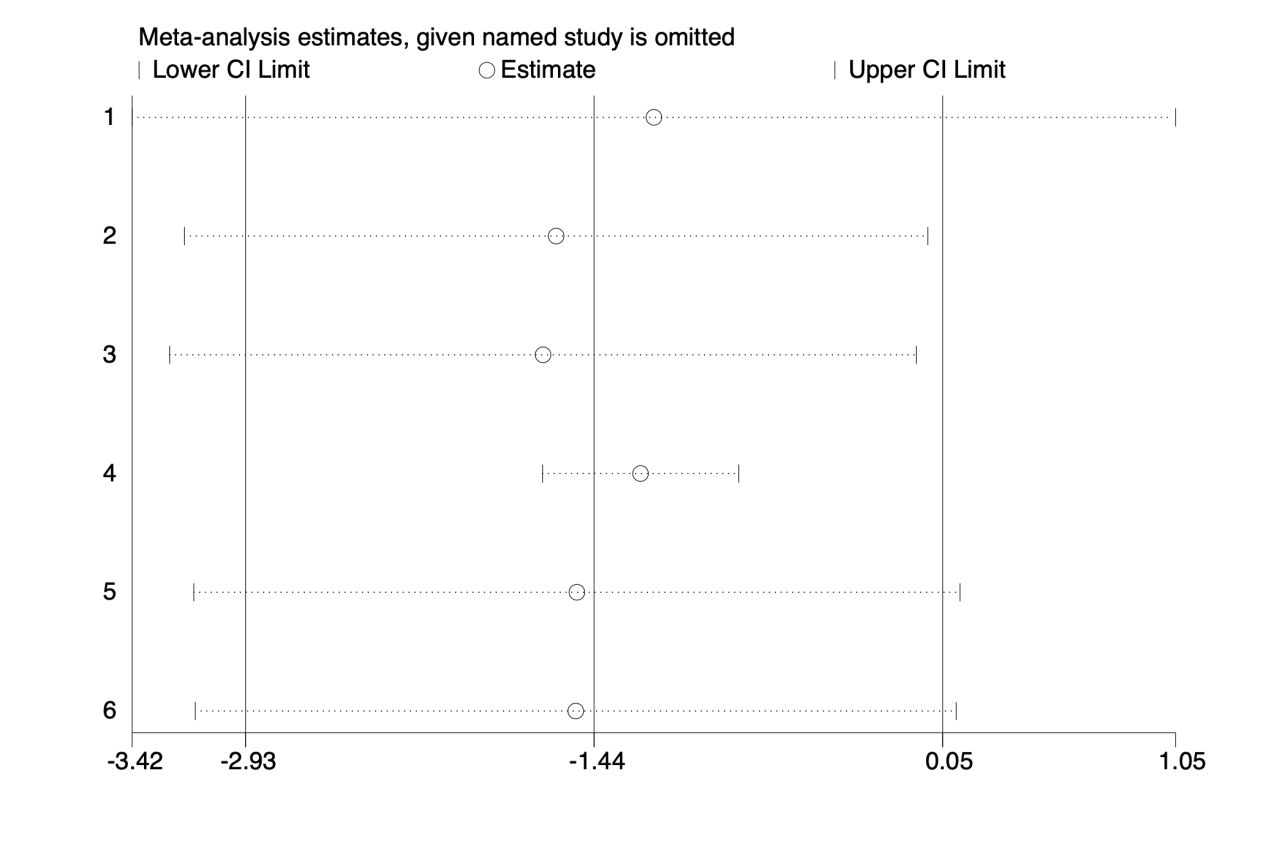


**Fig. S10** Sensitivity analysis of TBF


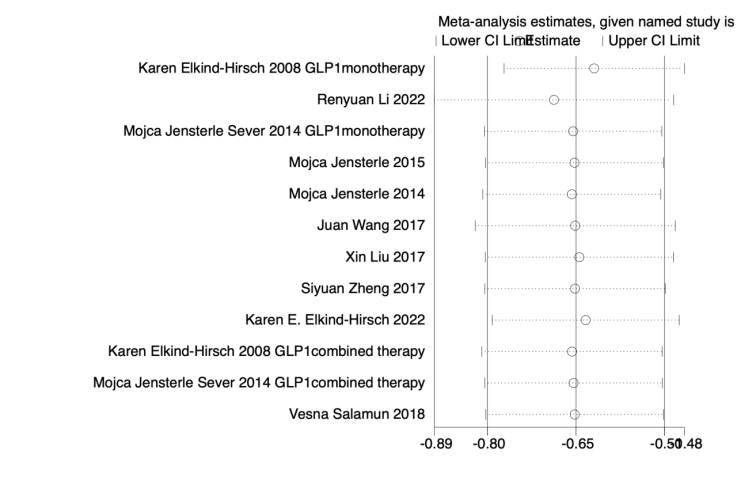


**Fig. S11** Sensitivity analysis of HOMA-IR


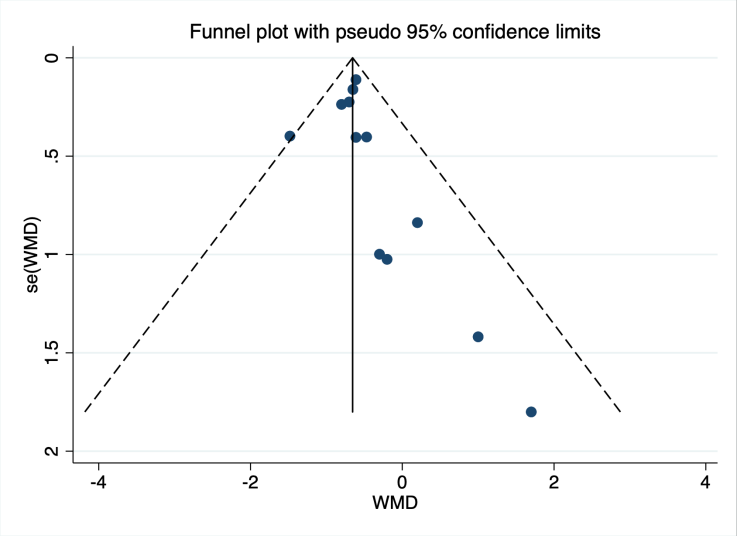


**Fig. S12** Funnel plot of HOMA-IR


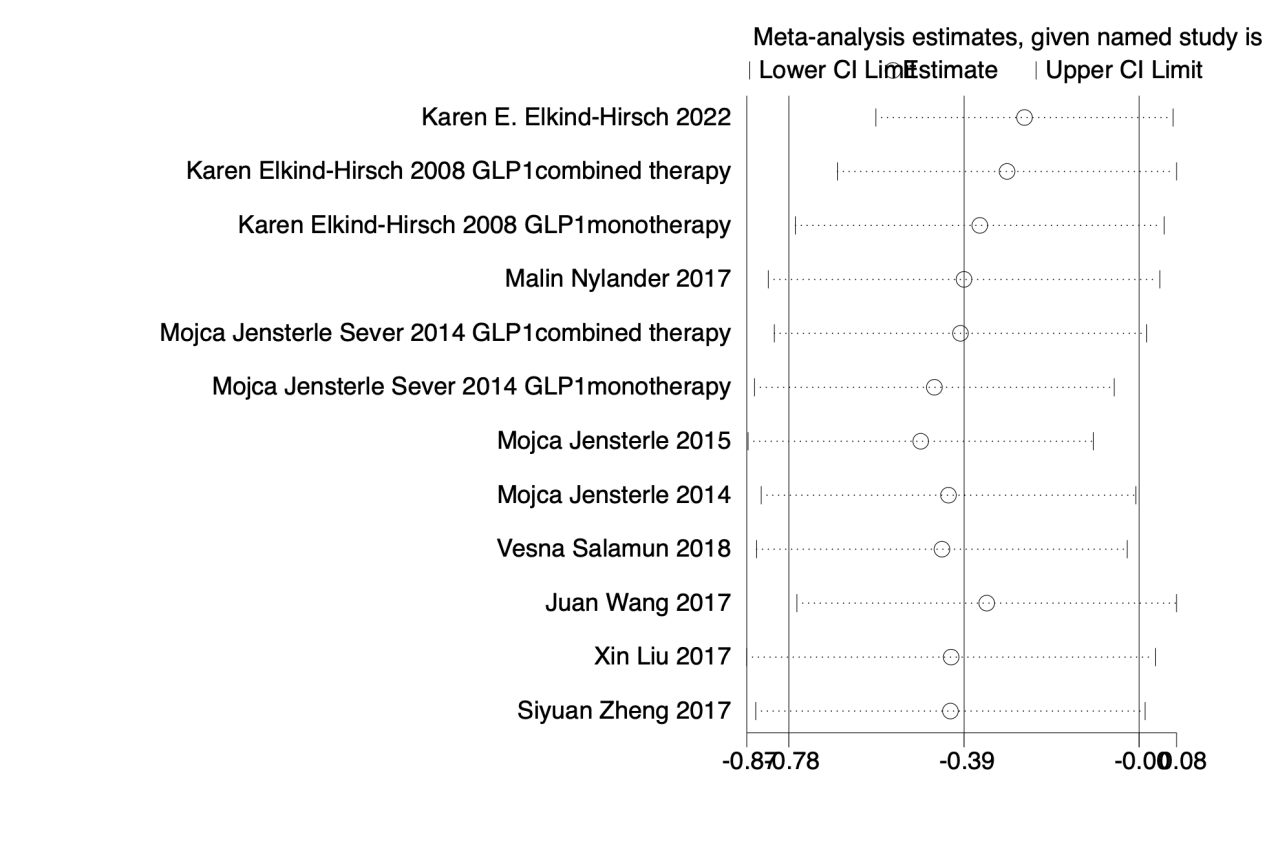


**Fig. S13** Sensitivity analysis of TT


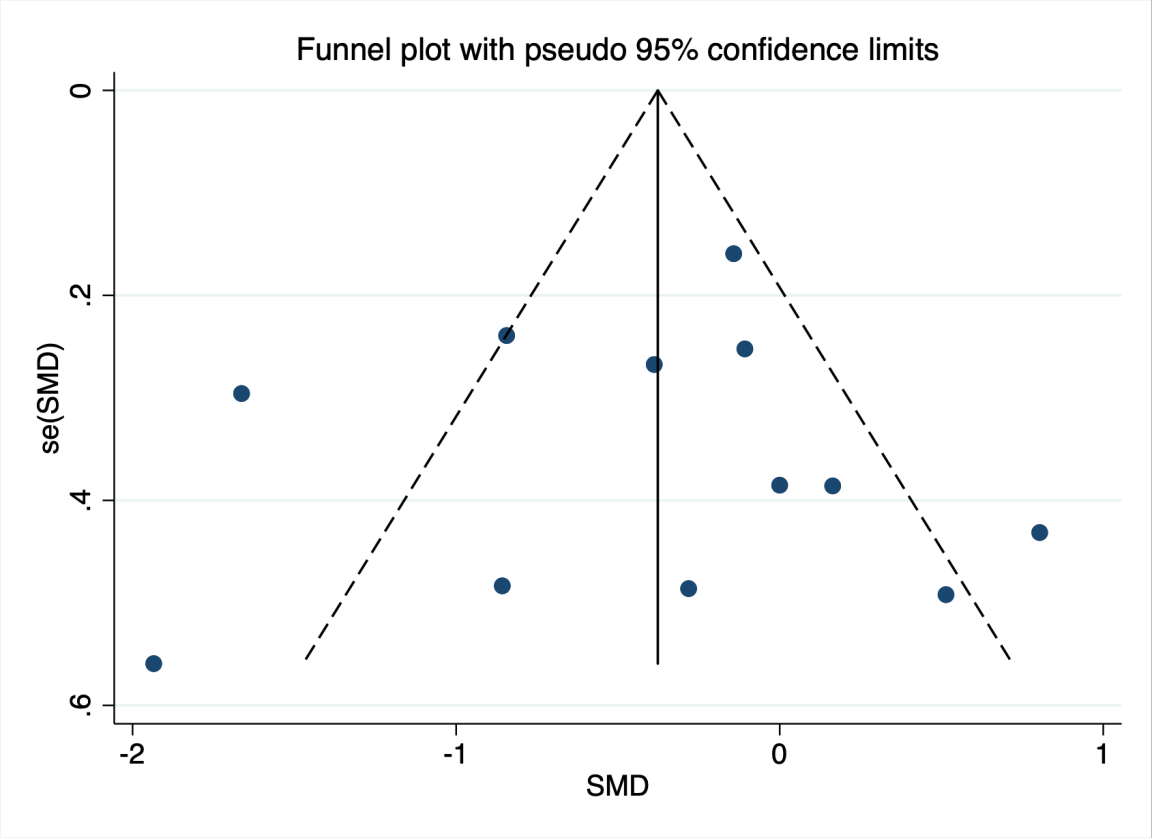


**Fig. S14** Funnel plot of TT


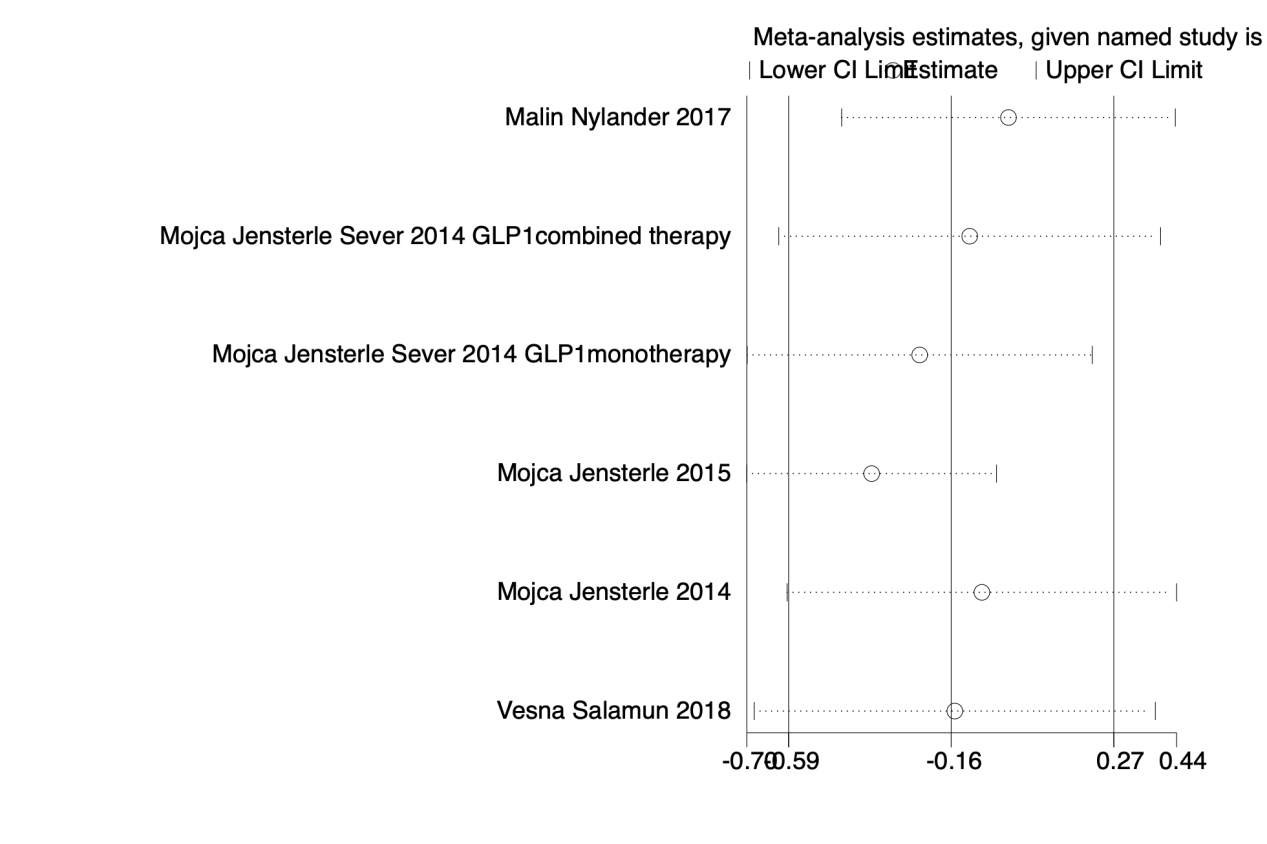


**Fig. S15** Sensitivity analysis of fT


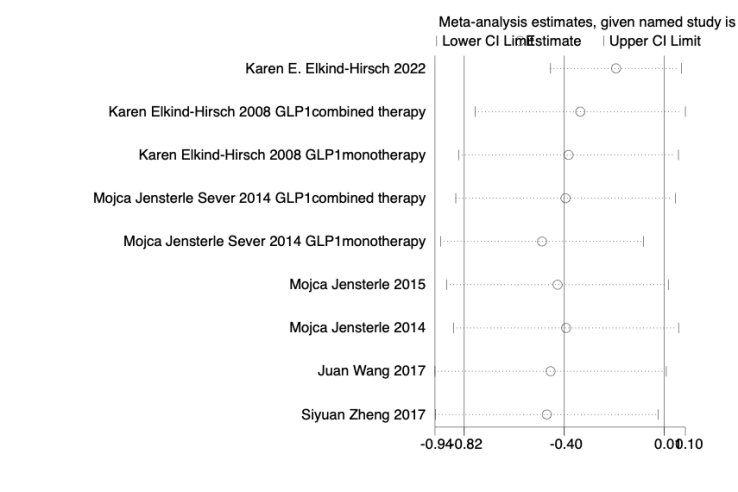


**Fig. S16** Sensitivity analysis of DHEAS


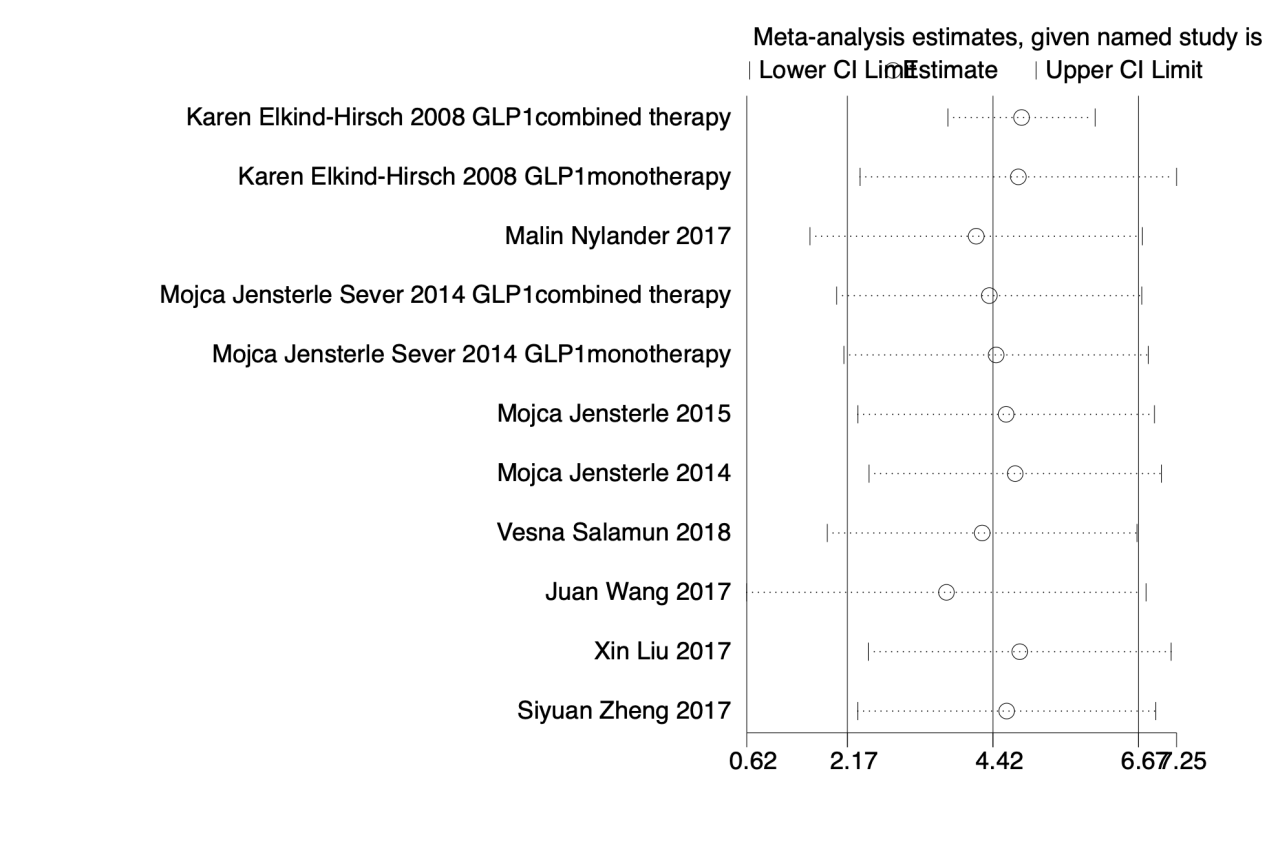


**Fig. S17** Sensitivity analysis of SHBG


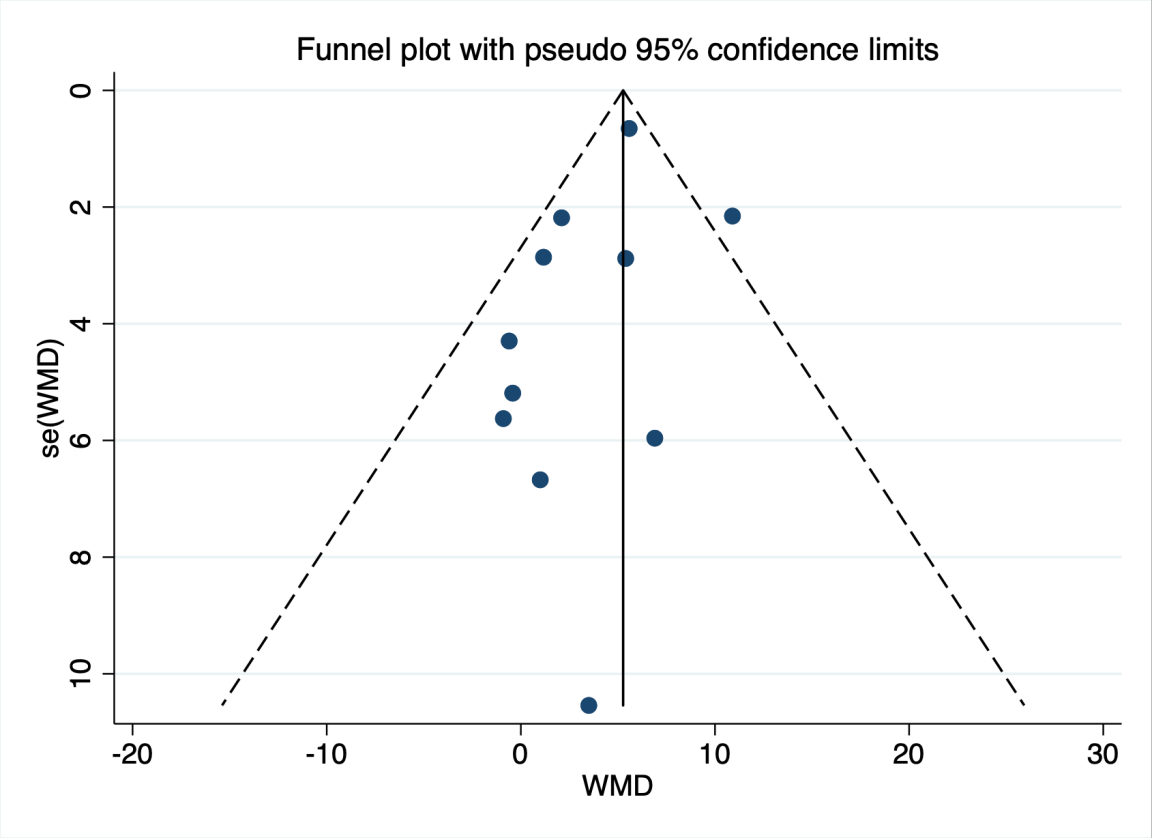


**Fig. S18** Funnel plot of SHBG


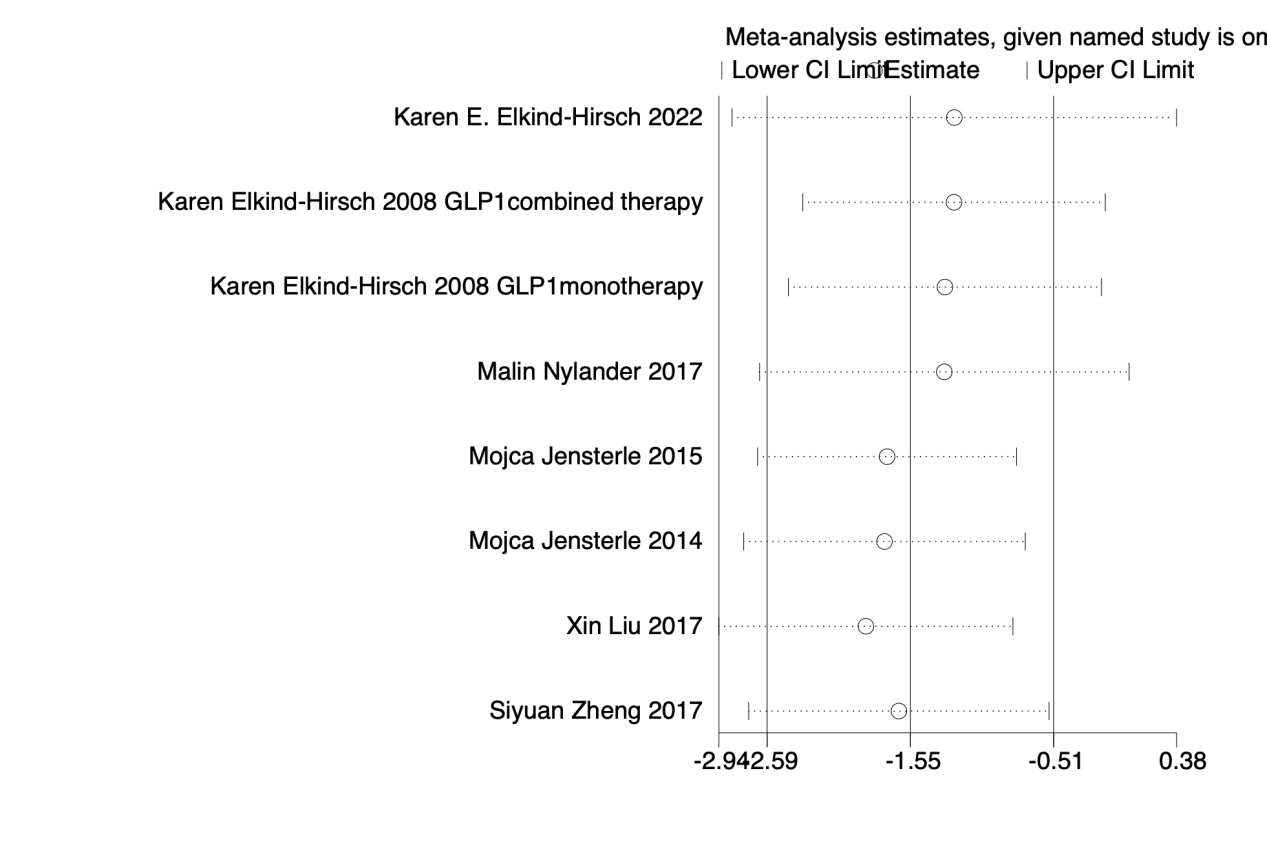


**Fig. S19** Sensitivity analysis of FAI
